# A Longitudinal Study of *Streptococcus pneumoniae* Carriage in a Cohort of Infants and Their Mothers on the Thailand-Myanmar Border

**DOI:** 10.1371/journal.pone.0038271

**Published:** 2012-05-31

**Authors:** Paul Turner, Claudia Turner, Auscharee Jankhot, Naw Helen, Sue J. Lee, Nicholas P. Day, Nicholas J. White, Francois Nosten, David Goldblatt

**Affiliations:** 1 Shoklo Malaria Research Unit, Mae Sot, Thailand; 2 Mahidol-Oxford Tropical Medicine Research Unit, Bangkok, Thailand; 3 Centre for Tropical Medicine, University of Oxford, Oxford, United Kingdom; 4 Immunobiology Unit, Institute of Child Health, University College London, London, United Kingdom; Centers for Disease Control & Prevention, United States of America

## Abstract

**Background:**

Pneumococcal disease is a major cause of childhood death. Almost a third of the world's children live in Southeast Asia, but there are few data from the region on pneumococcal colonization or disease. Our aim was to document the dynamics of pneumococcal carriage in a rural SE Asian birth cohort.

**Methods:**

We studied 234 Karen mother-infant pairs in Northwestern Thailand. Infants were followed from birth and nasopharyngeal swabs were taken from mother and infant at monthly intervals until 24 months old.

**Results:**

8,386 swabs were cultured and 4,396 pneumococci characterized. Infants became colonized early (median 45.5 days; 95% confidence interval [CI] 44.5-46.0) and by 24 months had a median of seven (range 0–15) carriage episodes. Maternal smoking and young children in the house were associated with earlier colonization (hazard ratio [HR] 1.5 (95% CI 1.1–2.1) and 1.4 (95% CI 1.0–1.9)). For the four commonest serotypes and non-typeable pneumococci, previous exposure to homologous or heterologous serotypes resulted in an extended interval to reacquisition of the same serotype. Previous colonization by serotypes 14 and 19F was also associated with reduced carriage duration if subsequently reacquired (HR [first reacquisition] 4.1 (95% CI 1.4–12.6) and 2.6 (1.5–4.7)). Mothers acquired pneumococci less frequently, and carried them for shorter periods, than infants (acquisition rate 0.5 vs. 1.1 /100 person-days, p<0.001; median duration 31.0 vs. 60.5 days, p = 0.001). 55.8% of pneumococci from infants were vaccine serotypes (13-valent pneumococcal conjugate vaccine, PCV13), compared with 27.5% from mothers (p<0.001). Non-typeable pneumococcal carriage was common, being carried at least once by 55.1% of infants and 32.0% of mothers.

**Conclusions:**

Pneumococcal carriage frequency and duration are influenced by previous exposure to both homologous and heterologous serotypes. These data will inform vaccination strategies in this population.

## Introduction

*Streptococcus pneumoniae* is estimated to be responsible for ∼10% of deaths in children aged <5 years [1]. Since almost a third of the world's children live in Southeast Asia, this pathogen is likely to be of considerable importance but there is a paucity of data on pneumococcal disease in the region [1]–[3]. Infant immunization with pneumococcal conjugate vaccines (PCV) is now routine in many countries including those that are GAVI (Global Alliance for Vaccines and Immunization) eligible [4]. In addition to the prevention of invasive disease, pneumonia, otitis media and all-cause mortality, PCVs have been shown to reduce colonization by the serotypes covered by the vaccine which has led to adaptive alterations in the serotypes colonizing the nasopharynx and causing disease [5], [6]. Surveillance of colonization is an important component of the vaccination monitoring process and robust pre-vaccine era data are important in planning and assessment of impact [7]. Given the large number of serotypes and many potential modifying agents, including other nasopharyngeal colonizers, respiratory virus infection, infant immune system maturation and antibiotic use, pneumococcal colonization dynamics are best understood from longitudinal data [8]–[23]. Such studies are complicated to perform so few have been comprehensive with regard to follow-up duration, consistency of sampling intervals, and clinical data collection to permit assessment of potential modifiers of pneumococcal carriage. Various sampling and culture methods have been described, adding to the heterogeneity between studies and prompting the development of a World Health Organization (WHO) standard protocol for pneumococcal nasopharyngeal colonization detection [24].

To explore colonization dynamics and modifiers, we designed an intense pneumococcal carriage study of mother-infant pairs from birth for 24 months. We nested the study within a longitudinal study designed to establish the epidemiology and etiology of pneumonia in a cohort of refugee children aged <2 years. We were particularly interested to understand the behavior of serotypes included in the current conjugate vaccines, as they are the most prevalent serotypes causing disease and conjugate vaccines are known to interfere with pneumococcal transmission by perturbing nasopharyngeal colonization.

**Figure 1:**
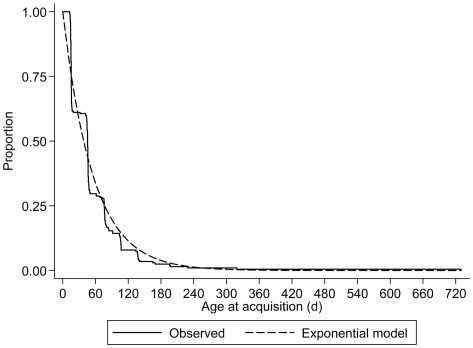
Observed and modeled age at first pneumococcal acquisition in the cohort of infants.

**Figure 2:**
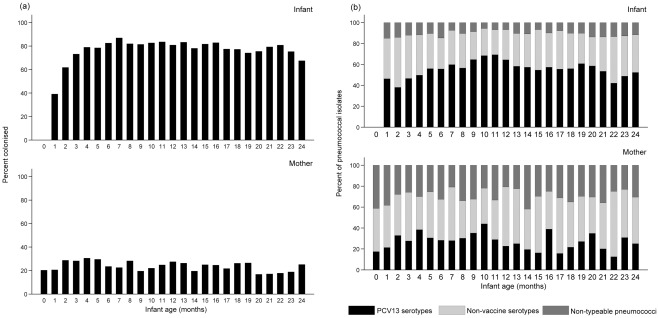
(a) Cross-sectional pneumococcal carriage prevalence and (b) pneumococcal isolates by serotype category (PCV13 serotype; non-vaccine serotype; non-typeable) in infants (top) and mothers (bottom) by month of infant age.

## Methods

### Study site and population

The study was carried out in Maela, a long-term camp for displaced persons in rural Northwest Thailand. Maela has a population of ∼40,000 displaced persons from Myanmar, predominantly of Karen ethnicity, who live in a 4 km^2^ area. Camp residents receive WHO EPI (Expanded Program on Immunization) immunizations. *Haemophilus influenzae* type B (HiB) and pneumococcal vaccines are not available. Shoklo Malaria Research Unit (SMRU) has provided medical care in this population since 1986.

Between October 2007 and November 2008, all pregnant women attending the SMRU antenatal clinic at 28–30 weeks gestation were invited to consent to their infant's participation in a pneumonia cohort study. Using sealed opaque envelopes containing an allocation code, women were randomly allocated to the pneumococcal carriage sub-cohort at enrolment. For this sub-cohort, women had a nasopharyngeal swab (NPS) taken at delivery and both infant and mother had a NPS taken at monthly surveillance visits from 1–24 months of age. Samples of blood were collected from the mother and umbilical cord at delivery and infant blood samples were taken at each monthly visit.

**Table 1:** Age at acquisition and duration of pneumococcal carriage (ten commonest serotypes, ranked by number of carriage episodes).

Infant: first episode				Infant: all subsequent episodes			Mother: all episodes		
Serotype	No. carriage episodes	Acquisition age, days Median (95% CI)	Carriage duration, days Median (95% CI)	Serotype	No. carriage episodes	Carriage duration, days Median (95% CI)	Serotype	No. carriage episodes	Carriage duration, days Median (95% CI)^a^
**NT**	35	46.0 (16.0–48.0)	31.0 (30.5–61.0)	**NT**	160	33.5 (31.5–60.0)	**NT**	145	31.5 (31.0–50.5)
**19F**	23	47.0 (15.5–79.5)	212.5 (77.5–242.5)	**19F**	146	91.5 (62.0–120.0)	**19F**	50	30.5 (30.0–31.0)
**23F**	21	44.5 (15.0–47.0)	184.0 (61.5–241.0)	**23F**	114	89.5 (62.0–94.0)	**23F**	31	31.0 (30.0–31.5)
**6B**	14	44.0 (15.5–48.0)	119.5 (60.5–152.5)	**6B**	105	62.0 (58.0–91.0)	**34**	25	31.0 (30.0–59.0)
**35F**	9	45.5^b^	121.0 (29.5–179.5)	**14**	72	86.5 (61.0–92.0)	**3**	23	45.5 (30.5–90.0)
**11A**	8	45.5 (15.5–77.0)	60.0 (30.0–89.5)	**15B/C**	68	60.5 (48.5–90.5)	**6B**	23	31.0 (29.5–31.5)
**14**	8	16.0 (12.5–83.0)	61.5(30.0–151.0)	**6A**	60	63.0 (47.5–120.5)	**14**	22	31.5 (30.5–59.5)
**28F**	8	45.5 (13.5–137.5)	60.5 (30.0–62.5)	**6C**	46	62.0 (33.0–93.5)	**11A**	18	32.5 (29.5–48.0)
**4**	7	15.5 (13.0–19.5)	83.5^b^	**19A**	43	58.5 (31.0–62.5)	**35C**	18	31.5 (30.0–32.5)
**34**	5	15.5^b^	122.5^b^	**34**	38	63.0 (31.5–134.0)	**15B/C**	16	31.5 (29.0–32.0)
**PCV13**	95	44.0 (16.0–46.5)	116.0 (88.0–127.0)	**PCV13**	611	62.0 (61.0–75.5)	**PCV13**	223	31.0 (31.0–31.5)
**NVT** c	95	45.5 (44.0–46.0)	61.0 (57.0–86.5)	**NVT** c	508	45.5 (32.5–57.5)	**NVT** c	316	31.0 (31.0–31.5)
**All**	225	45.5 (43.5–46.0)	63.0 (60.5–89.5)	**All**	1,279	60.0 (57.0–60.5)	**All**	684	31.0 (31.0–31.5)

a Duration calculated for 602 carriage episodes (acquisition date could not be calculated for carriage episodes including the first or second swab).

b Unable to estimate 95% CI.

c Excluding non-typeable (NT) pneumococci.

### Sampling and laboratory procedures

NPS were collected according to the WHO pneumococcal colonization detection protocol [24], [25]. Briefly, a Dacron-tipped swab (Medical Wire & Equipment) was used to sample the nasopharynx and the tip was excised immediately into a cryovial containing 1ml skim milk-tryptone-glucose-glycerol (STGG) medium. NPS-STGG specimens were transferred to the laboratory in a cool box and frozen at −80°C until culture, within eight hours of collection. Ten microliters of thawed STGG was cultured onto sheep blood-CNA agar (bioMerieux) and incubated overnight at 36°C in 5% CO_2_. Morphologically distinct alpha-hemolytic colonies were sub-cultured onto plain sheep blood agar. In the absence of morphologic variation, two representative colonies were sub-cultured. *S. pneumoniae* was identified by colonial morphology and optochin disc susceptibility (Oxoid). Isolates with reduced optochin disc zone diameters (7–13 mm) were confirmed as pneumococci by the bile solubility test [26]. Pneumococci were serotyped by latex agglutination with Quellung confirmation if this was equivocal [27], [28]. Serotype 6C was identified by PCR [29]. Isolates found to be non-typeable (NT) by latex agglutination were confirmed by bile solubility and absence of capsular swelling with Omniserum (SSI Diagnostica) [30].

### Definition of carriage

A carriage episode was defined as the period of time between acquisition and clearance of a pneumococcal serotype. Acquisition was identified when a pneumococcal serotype was cultured from a swab for the first time or when a serotype was re-cultured following clearance as defined below. Carriage episodes commenced at the midpoint between the last negative swab and the first positive swab for the serotype. Following acquisition, clearance of the serotype from the nasopharynx was considered to have occurred when two consecutive swabs were culture negative for that serotype. Termination of the carriage episode was defined as the midpoint between the last positive swab and the first negative swab for the serotype (Figure S1) [21]. Given the lability of their capsules, serotypes 15B and 15C were considered as a single serotype [31].

**Table 2:** Effect of previous carriage on reacquisition and carriage duration of common pneumococcal serotypes, controlling for age and carriage of heterologous serotypes (comparing first episodes of serotype carriage, not necessarily the infant's first ever carriage episode, with subsequent episodes of carriage of the same serotype).

Serotype	No. carriage episodes	Reacquisition number	Carriage duration		Time to reacquisition^b^	
	(first/reacquisitions)		HR (95% CI)^a^	*P*	HR (95% CI)^c^	*P*
**NT**	195 (129/66)	1^st^	1.13 (0.79–1.63)	.5	0.18 (0.10 –0.35)	< .001
		2^nd^	0.75 (0.33–1.71)	.5	0.22 (0.07–0.63)	.005
		3^rd^	0.31 (0.04–2.46)	.3	0.40 (0.12–1.38)	.1
**19F**	169 (112/57)	1^st^	2.64 (1.50–4.67)	.001	0.39 (0.14–1.08)	.07
		2^nd^	2.81 (1.09–7.22)	.03	0.35 (0.09–1.32)	.1
		3^rd^	1.39 (0.90–2.16)	.1	1.00 (0.19–5.16)	1.0
**23F**	135 (101/34)	1^st^	1.56 (0.94–2.60)	.08	0.50 (0.19–1.31)	.2
		2^nd^	3.20 (1.93–5.28)	< .001	0.19 (0.04–0.95)	.04
		3^rd^	0.76 (0.51–1.11)	.2	0.79 (0.08–7.99)	.8
**6B**	119 (85/34)	1^st^	1.18 (0.62–2.23)	.6	0.30 (0.11–0.84)	.02
		2^nd^	1.21 (0.59–2.46)	.6	0.21 (0.05–0.84)	.03
		3^rd^	-	-	-	-
**14**	80 (71/9)	1^st^	4.12 (1.35–12.59)	.01	0.04 (0.04–0.35)	.004
		2^nd^	-	-	-	-
		3^rd^	-	-	-	-

a Cox proportional hazards model.

b Time from clearance of a serotype to subsequent reacquisition.

c Parametric survival model (Weibull distribution).

### Data analysis

Data were entered into an Access 2003 database (Microsoft) and the entire database was manually checked for errors by comparing with the original case record forms. Statistical analyses were carried out using Stata/IC 12.0 (StataCorp). Comparisons were made using the Wilcoxon rank-sum test. Categorical data were analyzed using the chi-squared test. Logistic regression models, with either study participant identifier included as a random-effect or robust standard errors to control for repeated observations within individuals, were used to determine temporal changes in serotype distributions, multiple serotype detection, and transmission. Odds ratios, adjusted for the number of NPS specimens per individual, were calculated to determine the likelihood of carriage of serotypes in infants or mothers. Time to pneumococcal acquisition and carriage duration were estimated by survival analysis, since some carriage episodes were censored. The log-rank test was used to compare groups. Survival models (Cox proportional hazards or parametric models using exponential or Weibull distributions) were used to assess potential predictors of acquisition and carriage duration [32]. Model fit was assessed by examination of predicted Cox-Snell residuals, the Akaike information criterion (AIC), and log-likelihood values.

### Ethics

Written informed consent was obtained from the mothers prior to study enrolment. Ethical approval was granted by the ethics committees of the Faculty of Tropical Medicine, Mahidol University, Thailand (MUTM-2009-306) and Oxford University, UK (OXTREC-031-06).

**Figure 3:**
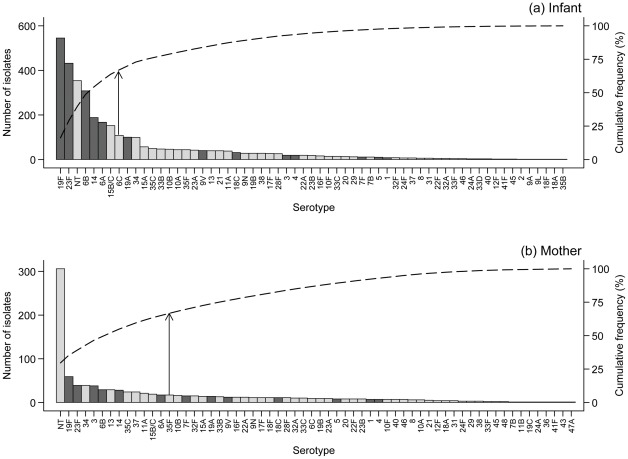
Pneumococcal serotype distribution in (a) infants and (b) mothers. The bars indicate the number of isolates of each serotype and the dashed lines indicate the cumulative frequency (cumulative frequency of 67% is indicated by the vertical arrows). Dark gray bars highlight PCV13 serotypes.

**Table 3:** Pneumococcal serotypes most commonly carried in infants and their mothers (ranked by overall isolation frequency).

Serotype	Overall N (%; rank)	Infant N (%; rank)	Mother N (%; rank)	Carriage in infants vs mothers OR (95% CI)^a^
**NT**	660 (15.0; 1)	354 (10.5; 3)	306 (29.6; 1)	2.8 (1.9–4.2)
**19F**	604 (13.7; 2)	545 (16.2; 1)	59 (5.7; 2)	4.5 (2.9–7.0)
**23F**	471 (10.7; 3)	432 (12.9; 2)	39 (3.8; 3)	6.3 (3.8–10.2)
**6B**	337 (7.7; 4)	308 (9.2; 4)	29 (2.8; 5)	7.3 (4.2–12.8)
**14**	216 (4.9; 5)	188 (5.6; 5)	28 (2.7; 6)	4.7 (2.7–8.0)
**6A**	184 (4.2; 6)	167 (5.0; 6)	17 (1.7; 10)	5.2 (2.8–9.7)
**15B/C**	171 (3.9; 7)	152 (4.5; 7)	19 (1.8; 9)	5.7 (3.2–10.4)
**34**	138 (3.1; 8)	99 (2.9; 10)	39 (3.8; 3)	1.8 (1.0–3.2)
**6C**	117 (2.7; 9)	108 (3.2; 8)	9 (0.9; 18)	6.7 (3.0–14.7)
**19A**	114 (2.6; 10)	100 (3.0; 9)	14 (1.4; 13)	3.9 (2.0–7.6)
**PCV13 serotypes**	2,162 (49.2%)	1,878 (55.8%)	284 (27.5%)	9.0 (5.1–15.7)
**NVT serotypes** b	1,574 (35.8%)	1,131 (33.6%)	443 (42.9%)	8.3 (4.4–15.6)
**Total**	4,396	3,363	1,033	4.9 (2.3–10.2)

a OR for the serotype being carried at any time point (adjusted for number of swabs collected per individual; all *P*<.05).

b Excluding non-typeable (NT) pneumococci.

## Results

Of 999 pregnant women recruited into the pneumonia cohort study, 250 were randomly allocated to the pneumococcal carriage sub-cohort. Nineteen women and their babies did not attend the first follow up visit (14 lost to follow up, four neonatal deaths, and one stillbirth) resulting in 231 mothers and 234 infants (three sets of twins) who had at least one surveillance NPS collected. These 234 mother-infant pairs are included in the analysis.

8,386 NPS were collected (73.6% of expected): 4,195 from mothers and 4,191 from infants (median 23 per individual; range 1–24 [infants], 2–25 [mothers]). The median mother-infant pair follow-up duration was 23.9 months post-delivery (728 days; range 28–760). Overall there were 2,188 carriage episodes identified (1,504 infant; 684 mother): infants had a median of seven (range 0–15) carriage episodes, compared with a median of two (range 0–16) in mothers (*P*<.001).

### Acquisition dynamics in infants

Infant acquisition was extremely rapid and by the three month visit, 75.7% of infants had been colonized increasing to 97.0% by six months. All but one infant had acquired pneumococci at least once by the eleven month visit. The median age of first acquisition was 45.5 days (95% confidence interval [CI], 44.5–46.0), with an observed and modeled acquisition rate of 1.8 per 100 child-days (Figure 1). Carriage stabilized after seven months and point-prevalence remained between 67.6–83.6% (Figure 2a). Twenty percent of mothers were colonized by pneumococcus at delivery (+/− 7d). This proportion increased after birth, peaking at 30.6% at the four month visit and subsequently stabilizing with monthly colonization point-prevalence of 16.8–29.6% (Figure 2a).

We explored whether age at first acquisition was associated with household size, presence of other young children in the house, ethnic group, prematurity (delivery <37 weeks gestation), home delivery, season of birth, maternal pneumococcal colonization at birth, maternal smoking, or antibiotic consumption in the neonatal period (Table S1). We could not assess the impact of feeding differences on colonization age since breast feeding was almost universal. By univariate analysis, large households (>5 people), other children <5y in the house, mothers who smoked, and home delivery were all associated significantly with earlier acquisition age. In a multivariate Cox model, the presence of other young children and maternal smoking remained associated significantly with an earlier age at first colonization (children <5y: hazard ratio [HR] 1.4, *P* = .03; maternal smoking: HR 1.5, *P* = .01). Age at first acquisition was not significantly different between the commonest eight serotypes or between grouped vaccine (PCV13) serotypes, non-vaccine serotypes (NVT), and non-typeable (NT) pneumococci (Table 1).

### Pneumococcal carriage kinetics

The median duration of the infants' first ever carriage episode was 63.0 days (95% CI, 60.5–89.5). Duration of carriage of serotypes 19F and 23F was longer (*P*<.001 and .008 respectively), and NT pneumococci shorter (*P* = .01), than for the other serotypes (Table 1). Following the first carriage episode, infants had 1,279 subsequent pneumococcal acquisitions: 620 in the first year of life and 659 in the second. NT pneumococci, 19F, 23F, and 6B remained the most commonly carried serotypes (Table 1). Median age at second pneumococcal serotype acquisition was 111.0 days (95% CI, 107.0–137.0), with no significant differences between PCV13 serotypes, NVT, and NT pneumococci. The median interval between acquisitions was 62.0 days (95% CI, 61.0–62.5) and there was no difference in acquisition rates between PCV13 serotypes, NVT, and NT pneumococci. Considering all subsequent carriage episodes, PCV13 serotypes were carried for longer than NVT and NT pneumococci (medians 62.0, 45.5, and 33.5; *P*<.001), although there was substantial variation between individual serotypes (Table 1).

There were 272 instances of re-acquisition of a previously carried serotype. NT pneumococci, 19F, 23F, 6B and 14 accounted for ∼75% of these re-acquisitions. Controlling for age and carriage of heterologous serotypes, previous carriage of serotype 14 or 19F was associated with significantly reduced duration (HR>1) of subsequent carriage episodes of the same serotype, and there was a trend in the same direction for serotype 23F. Previous carriage was also associated with an increased interval to reacquisition (HR<1) of the same serotype (Table 2). Previous colonization by heterologous serotypes also impacted on time to reacquisition of these five serotypes (HR<1, *P*<.05 for all serotypes), but only for 19F could a reduction of carriage duration be demonstrated (HR 2.01, *P* = .01).

The pneumococcal nasopharyngeal acquisition rate was significantly lower in mothers compared with infants (0.5 vs. 1.1 per 100 person-days; *P*<.001). Mothers carried pneumococci for a shorter duration than infants (median 31.0 days vs. 60.5; *P*<.001) (Table 1).

There were 3,963 mother-infant swab pairs (swabs taken from both mother and infant on the same day), and in 233 (5.9%) a common serotype was identified. Identification of a common serotype in a mother-infant pair became less common as infant age increased (*P* = .02) which, along with an increasing proportion of colonized mothers in the four months following delivery, suggests transmission occurred more frequently in the early months of life.

### Pneumococcal serotype distribution

A total of 4,396 pneumococci were isolated from all swabs taken, comprising 67 serotypes (3,363 [infants]; 1,033 [mothers]). In infants, eight serotypes (19F, 23F, NT, 6B, 14, 6A, 15B/C, 6C) accounted for 67.0% of the isolates (Figure 3a). In mothers, 29.6% of isolates were non-typeable with no other predominant serotypes (Figure 3b). 55.8% of pneumococci from infants were PCV13 serotypes, compared with 27.5% from mothers (*P*<.001) (Table 3). The proportion of pneumococci that were PCV13 serotypes increased significantly over the first year of life in infants (46.2% [1 month] to 64.4% [12 months]; *P*<.001) and then decreased over the second year (Figure 2b). Infants carried a median of five (range 0–13), and mothers a median of two (range 0–11), serotypes over the 24 month observation period. Considering serotype carriage over the entire follow-up period (as binary “yes/no” variables for carriage of each serotype in an individual), all of the most common serotypes were more likely to be carried by infants than mothers, controlling for the number of NPS collected per individual in a logistic regression model (OR >1, Table 3).

In swabs where pneumococci were cultured, multiple serotypes were identified in 5.1% of infant swabs and 2.6% of mother swabs. Detection of more than one carried serotype became more common as age increased (infants *P*<.001; mothers *P* = .001).

## Discussion

This is the largest longitudinal pneumococcal carriage study in children under two years, in terms of the number studied and the sampling frequency/duration of follow up. Adherence to the WHO detection protocol enables direct comparison with other similar studies. The high level of follow-up and the uniformity of swab frequency over the follow-up period permitted confident assessment of carriage prevalence and dynamics. The inclusion of consistent follow-up in the second year of life was important since the risk of invasive disease remains high [33], [34], but there is scant detailed data on pneumococcal carriage dynamics in children aged 12–24 months. We found that 43.8% of all observed pneumococcal acquisitions occurred in the second year of life and that there were significantly more re-acquisitions of previously carried serotypes in the second 12 months compared to the first 12 months of life, perhaps as a result of waning immunity.

In this crowded refugee camp, infants were colonized frequently by pneumococci and the first acquisition occurred early in life. The median age of acquisition (45.5 days) is similar to published studies from Asia and Africa, although in individual studies from Papua New Guinea and The Gambia, infants were colonized even earlier (60% by 15 days and 50% by 33 days respectively) [9], [21]. In contrast, studies from the USA and Europe document older age at first pneumococcal acquisition, and lower overall carriage prevalence: the mean age at first acquisition was six months in the USA [8], and 56% of Finnish infants were colonized by 12 months with an overall carriage prevalence of 21% in the first two years of life [16]. In the current cohort, having a mother who smoked or other young children in the household was associated with an earlier age at first pneumococcal colonization. Siblings have been previously recognized as a risk factor for pneumococcal acquisition [12]. The presence of smokers in the household has not been consistently identified as a risk factor for infant pneumococcal carriage, although infants in India who were exposed to ≥20 cigarettes per day were at increased risk of colonization at two months of age [15].

Carriage duration varied by serotype, with serotypes 19F and 23F being carried for the longest periods in infants. For serotypes 14 and 19F, previous carriage of the serotype was associated with significantly increased interval to reacquisition and shorter duration of subsequent carriage of the same serotype. This trend was observed for 23F, although the reduction in carriage duration did not reach significance. These findings suggest that nasopharyngeal exposure to serotypes with immunogenic capsular polysaccharides (14/19F > 23F > 6B) result in an immune response capable of enhancing clearance if reacquisition occurs. The fact that NT carriage was also associated with delayed reacquisition of NT pneumococci implies that other factors, such as antibody responses to common surface proteins, are also involved in the regulation of pneumococcal nasopharyngeal colonization. Modeling of data from previous longitudinal carriage studies has demonstrated evidence for both serotype-dependant and independent effects on pneumococcal serotype acquisitions in young children, but not both in the same study population. Carriage of eight serotypes was modeled in Israeli infants aged 12–35 months, and a significantly lower risk of acquisition of serotypes 6A, 14, and 23F in those previously colonized was found [35]. Serotype-independent protection against reacquisition of four serotypes was shown in Bangladeshi infants aged <1 year [36].

Eight serotypes accounted for two-thirds of the pneumococcal isolates from infants. Serogroups 6, 19, 23 and serotype 14 have been the most prevalent pneumococci in the majority of previous infant/childhood carriage studies, regardless of geographical location [15], [16], [18], [21]. As expected, the proportion of PCV13 serotype carriage was greater in infants than in their mothers [17], [37], [38]. An unexpected finding was the high prevalence of non-typeable pneumococcal carriage. These organisms have limited disease potential, but may act as an important reservoir of antimicrobial resistance genes [39]. They are not consistently reported in carriage studies: they may not be identified because of atypical colony morphology or they may be actively excluded because of lack of association with invasive disease. When included, prevalence has varied from 0.7% of pneumococci in a Kenyan survey of adults and children to 44.5% in Spanish primary school children [38], [40]. In our study, these organisms were carried at least once by 55.1% of infants and 32.0% of mothers. The vast majority of isolates appeared non-encapsulated, rather than being pneumococci of a novel capsular type (data not shown). While it is possible that these organisms were misidentified non-pneumococcal streptococci or the result of poor quality serotyping quality control this is unlikely as all were confirmed by optochin susceptibility and bile solubility, and serotype was rechecked by Quellung. In addition 168 NT pneumococci from this collection have been genotyped by MLST and 96.4% were typed successfully, confirming their identity [41], [42]. Further characterization of a small sample of isolates by microarray-based comparative genomic hybridization and subsequent sequencing of the *cps* locus, confirmed their pneumococcal identity and demonstrated deletion or disruption of the capsule biosynthesis genes in 17/18 (94.4%) isolates [43].

There are some limitations to the study. Firstly, the WHO culture protocol underestimates the prevalence of multiple serotype carriage. We recently confirmed that while the predominant serotype is likely to be identified correctly, minor carried serotypes may be missed [25]. This may result in an underestimation of carriage duration. It may also explain the emergence of “replacement” serotypes seen when PCV is used and the predominant carriage serotypes decline [7]. Secondly, the sampling strategy we employed means that we only have limited data regarding adult pneumococcal carriage prevalence and transmission of serotypes within the household. The carriage prevalence in the mothers (24.0%) was higher than that documented in Kenyan adults (5.3%), similar to Papua New Guinean mothers (∼30%), but lower than Gambian adults (>50%) [9], [37], [38]. We elected to swab the mothers since they were likely to have the greatest contact with the infants and therefore offered a reasonable opportunity to study pneumococcal transmission. However, the large household sizes and the crowded nature of the camp inevitably resulted in the majority of pneumococcal transmission being unobserved. In spite of this we documented a trend in concordance of serotypes between mothers and their infants, suggesting that transmission was more frequent in the early months of life. The monthly interval between nasopharyngeal swabs will have resulted in the under detection of serotypes carried for short durations. This is particularly relevant to the estimation of carriage prevalence in adults, as median carriage duration was only 31 days (i.e. a serotype detected at a single swabbing point only) in the study mothers.

In conclusion, we have documented the characteristics of pneumococcal carriage in mothers and infants in a rural SE Asian community. These data will inform vaccination strategies in this population. Determination of timing of transmission of pneumococci between mother and infant, and the impact of early colonization by a serotype on subsequent acquisitions, improve understanding of the complex process of early pneumococcal nasopharyngeal colonization. These observations will be supported by on-going molecular and immunologic studies of the pneumococcal isolates and the cohort, illuminating molecular changes in pneumococci in response to carriage and defining immune responses to pneumococcal antigens that may identify targets for new pneumococcal vaccines.

## Supporting Information

Figure S1 **Carriage episode definition.** In this example, the individual first carries 6B and subsequently acquires 19F. Following clearance, there is reacquisition of serotype 6B.
(PDF)

Table S1 **Univariate and multivariate Cox regression analyses of potential factors affecting age at first ever pneumococcal acquisition in infants (Hazard Ratio >1 indicates earlier age at first acquisition).** All factors were included in the multivariate model.
(PDF)
