# Supplementary material for: A Longitudinal Study of Streptococcus pneumoniae Carriage in a Cohort of Infants and Their Mothers on the Thailand-Myanmar Border
Source: PLoS One. 2012 May 31;7(5):e38271. doi: 10.1371/journal.pone.0038271 (PMC3365031; doi:10.1371/journal.pone.0038271)
Supplement: Figure S1 — Carriage episode definition. In this example, the individual first carries 6B and subsequently acquires 19F. Following clearance, there is reacquisition of serotype 6B. (PDF) [file pone.0038271.s001.pdf]

Figure S1. Carriage episode definition.

In this example, the individual first carries 6B and subsequently acquires 19F. Following clearance, there is reacquisition of serotype 6B.

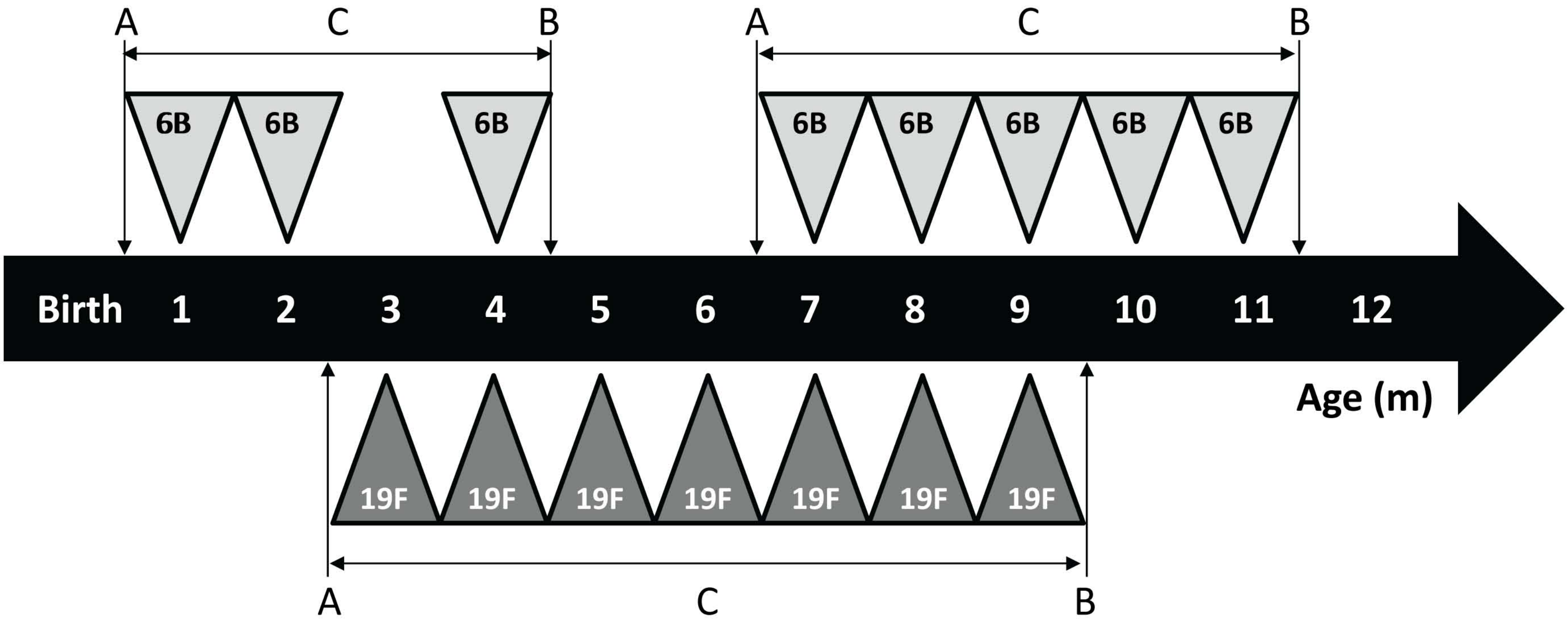

A. Acquisition (mid-point between last negative swab and first positive swab for a serotype)
B. Clearance (mid-point between last positive swab and first of two consecutive negative swabs for a serotype)
C. Carriage duration (clearance date – acquisition date)
