# Supplementary material for: A Longitudinal Study of Streptococcus pneumoniae Carriage in a Cohort of Infants and Their Mothers on the Thailand-Myanmar Border
Source: PLoS One. 2012 May 31;7(5):e38271. doi: 10.1371/journal.pone.0038271 (PMC3365031; doi:10.1371/journal.pone.0038271)
Supplement: Table S1 — Univariate and multivariate Cox regression analyses of potential factors affecting age at first ever pneumococcal acquisition in infants (Hazard Ratio >1 indicates earlier age at first acquisition). All factors were included in the multivariate model. (PDF) [file pone.0038271.s002.pdf]

Table S1. Univariate and multivariate Cox regression analyses of potential factors affecting age at first ever pneumococcal acquisition in infants (Hazard Ratio >1 indicates earlier age at first acquisition). All factors were included in the multivariate model.

**Factor**	**Univariate model**		**Multivariate model**	
	**Hazard Ratio (95% CI)**	***P***	**Hazard Ratio (95% CI)**	***P***
Household size >5 people	1.34 (1.02 – 1.76)	.04	1.16 (0.86 – 1.57)	.3
Children <5y in the house	1.53 (1.17 – 2.01)	.002	1.40 (1.03 – 1.89)	.03
Mother smoker	1.63 (1.21 – 2.19)	.001	1.52 (1.09 – 2.11)	.01
Ethnic group:				
Sgaw Karen	-		-	-
Pwo Karen	0.79 (0.51 – 1.24)	.3	0.87 (0.54 – 1.40)	.6
Muslim	1.12 (0.78 – 1.75)	.5	1.25 (0.81 – 1.92)	.3
Other	1.38 (0.56 – 3.36)	.5	1.73 (0.69 – 4.37)	.2
Season of birth:				
Hot (March – May)	-		-	-
Wet (June – October)	0.75 (0.52 – 1.06)	.1	0.81 (0.55 – 1.18)	.3
Cool (November – February)	1.08 (0.75 – 1.54)	.7	1.09 (0.74 – 1.59)	.7
Home delivery	1.56 (1.13 – 2.16)	.007	1.25 (0.86 – 1.80)	.2
Prematurity	0.61 (0.38 – 1.00)	.05	0.55 (0.30 – 1.00)	.05
Mother colonized at birth	1.28 (0.92 – 1.77)	.1	1.19 (0.84 - 1.69)	.3
Antibiotics in the neonatal period	0.79 (0.52 – 1.22)	.3	0.87 (0.54 – 1.42)	.6
